# Books and Magazines

**Published:** 1909-03

**Authors:** 


### Books and Magazines.

Agents wanted for Goldthwait, Painter and Osgood's, Cabot's, and Austin's books, and the *Boston Medical and Surgical Journal.* Write for special offer.

D. C. Heath & Co., Boston, Mass.

